# Cases in Surgery

**Published:** 1843-08

**Authors:** John M. Esselman

**Affiliations:** Nashville, Tennessee


Art. II.—*Cases in Surgery.*—Reported to the Medical Society of Tennessee. By John M. Esselman, M.D., of Nashville, Tennessee.

Case I.—*Complete Extirpation of the Uterus by Ligature, after Chronic Inversion of the Organ.*—On the 1st of September, 1834, I was requested by Mr. Moore, of this city, to visit his wife, a very intelligent lady, about thirty-two years of age. On arriving at his house, I learned the history of her case from herself and husband to be as follows: They had been married for fourteen years, and their only child, a daughter, was then about twelve years of age; Mrs. M. had been in bad health ever since the birth of their child. They resided in the county of Williamson at the time of her confinement, and not being near a physician she was attended by an old lady of the neighborhood, who was in the habit of officiating as midwife. She informed me that her delivery was a very protracted and painful one, even beyond that which is usual in giving birth to the first child. I questioned her particularly respecting the delivery of the placenta, or after birth; in answer, she recollected that the old lady had given her a great deal of pain, and that she had flooded profusely, and was very faint at the time; that she was very ill for several weeks after her confinement, and was attended by a physician from Franklin, whose name I do not recollect; and that after she was able to leave her bed and walk about the house, she was much annoyed by "*bearing down pains*," as she called them, in the region of the womb, extending up in the direction of the lateral ligaments of that organ. She informed me also that she had suffered much from pain and weakness of the back, and also from pain and a numb sensation down the inner portion of the thighs, and had been a prey to fluor albus ever since she had left her bed of confinement, with the exception of her catamenial periods, which are very irregular. She stated that the secretion was often very profuse, indeed alarmingly so; that she would be confined to her bed for weeks at a time, had to consult physicians, take medicine to check the hemorrhage, &c.; then she would be put on the use of tonics, to strengthen her system, as well as to correct the fluor albus. At length she was advised by her physicians that she was laboring under prolapsus of the womb, and underwent the routine of treatment in such cases, horizontal position, tonics, astringent injections, the use of the pessary, &c., but all to no effect, except the relief she invariably obtained from the horizontal position. Disheartened by repeated failures of various prescriptions, and almost exhausted by disease, she was advised to consult the late Dr. Purgsley of this city, who was thought to be very eminent in his profession, especially in the branch of surgery; the Dr. advised she should be removed to Nashville, saying upon examination she was laboring under polypus of the uterus. Accordingly she was brought in, and after some preparatory treatment, he applied the ligature, but it was attended with so much pain, that it was impossible for her to bear it; the pain was so severe as to produce fainting, vomiting, and even threatened convulsions. The symptoms were so alarming that the Doctor took off the ligature and abandoned the operation, but directed his attention to her general health, which was very much improved under his prescriptions for the time. Mr. Moore moved to

Louisville and resided there until the year 1834, at which time he returned to Nashville; during her absence she had consulted many medical men, but all to no purpose.

At the time I was consulted I found her in a deplorable situation: she was laboring under hectic fever, had profuse night sweats, hacking cough, and all the symptoms indicative of a rapid decline; on examination I found a tumour occupying the vagina, about the size of a large pear, and answering in every respect the description usually given of a polypus. The vagina itself was very irritable and much ulcerated, so that it was impossible to make a very minute or satisfactory examination. However, from the history of the case, and the opinion of other medical men who had examined it previous to myself, in some of whom I had the utmost confidence, I concurred with them in the opinion that it was a *polypus.* But being at that time a young practitioner, having been but two years in the profession, I requested that some other physician should be called in to assist me in the operation; Dr. Waters of this city, was called in, than whom no one is more intelligent or expert in the practice of surgery, and after an examination and a few days of preparatory treatment, we applied the ligature, made of saddler's silk well twisted and waxed, in the following manner: The patient was placed in the usual position for turning the child, one end of the ligature was passed through one of the tubes of a double canula, the other end was attached to the shoulder of the same tube. I took the canula in my right hand, and the end of the ligature in my left, and passed the canula up the vagina to the footstalk or pedicle of the polypus as we supposed; then I applied an instrument, which had suggested itself to my mind, formed out of the stay of a parasol (that supports the ribs) which is notched or forked at the end; I had the notched end filed very smooth and gave it a slight curve, to fit the course of the vagina, which formed a very delicate instrument well suited to the purpose we intended, which I shall call a *director.* After having passed the canula to the point above stated, I held it steady, and requested Dr. Waters to pass the director up the vagina with the ligature resting in the notched end, until it reached the same point of the canula. I then gave him the ligature to hold, and took the director in my left hand, having the canula in my right; brought the canula up on the right side of the tumour and the director up on the left, until the instruments met on its upper surface; then by passing the end of the canula over the notched end of the director, let the ligature fall into the notch or fork of the director. I then held the director fast, and retracted the canula outside of the vulva, passed the ligature up the other tube of the canula, then passed the canula again into the vagina, containing now as you perceive both ends of the ligature, up to the end of the director, which relieved it of the ligatures it contained in its notched or forked end, and completely encircled the neck of the tumour. I now withdrew the director, and made the other end of the ligature fast to the shoulder of the other tube of the canula, drawing the ligature as tight as we thought safe for the time. Although two hours before we commenced the operation we administered to her a full portion of spirits of camphor, hartshorn and laudanum, the tightening of the ligature gave her a great deal of pain, so much so that I repeated the stimulus and narcotic. I remained with the patient for four or five hours; at first she was very much prostrated, her pulse sunk to a mere thread. However, within four hours she became composed, reaction took place and she rested tolerably well the first night; I watched her closely and saw her frequently and tightened the ligature every morning for eighteen days, at which time it came away. You can readily imagine my surprise upon the examination of the tumour, to find it, instead of a polypus as was supposed by all who had inquired into the nature of the case, to be the uterus itself, although very much reduced in size by ulceration and strangulation. The vagina was much ulcerated and emitted a very offensive sanious discharge as I before mentioned. I directed frequent injections into the canal, of a solution of the chlorate of lime, as an antiseptic, and a solution of the nitrate of silver, as an application to the ulcers. I sustained the general system by the use of tonics, such as the muriated tincture of iron, phosphate of iron, quinine, &c., and a generous diet when the absence of febrile excitement would admit of it.

She was a long while recovering and did not leave her bed for months after the operation, but finally was restored to perfect health, and I venture to say, she is as fine a looking woman of her age as walks the streets of this city to day. She has never menstruated since, of course; I had to bleed her frequently for the first twelve months after her recovery, and give her purgatives to relieve headache and a tendency to vertigo, as well as a general plethora of the system, occasioned, I have no doubt, by the premature suspension of the catemenial secretion.

It is evident in this case that the uterus was inverted at the birth of her child, and that that was the prime cause of all her bad health and suffering for the twelve years previous to the operation.

You see gentlemen I have acknowledged the error I committed here in extirpating the uterus, when I supposed it to be a polypus. However, I have this apology to offer as an extenuation, that Dr. Newnham, an English surgeon, who has written a very learned essay on the subject of inverted uterus, frankly states that he extirpated a uterus, which he thought to be a large sized polypus, and this fact induced him to write and publish on the subject. Moreover all the medical gentlemen who had examined this case previous to myself, had given it as their opinion that it was polypus, and in fact the parts were in such a condition when I saw the patient, that it was impossible to make a minute examination. This case is written out from notes taken at the time I attended the patient. I am aware that it is imperfectly done, but the facts are set forth as they occurred, and that is the principal object, as I understand it, in reporting a case.

Case II.—*Acute Laryngitis Relieved by Tracheotomy.*—Captain Jesse Johnson, of the steamboat Water-Witch, was attacked with bilious pneumonia, at Smithland on the Ohio river, at which point he received medical aid, and was partially relieved; he arrived here on a boat, on the 12th of February, 1837, at which time I was requested to attend him. I found him with considerable fever, and irritation about his chest, distressing cough, expectoration streaked with blood; I bled him freely, gave him mercurial purgatives combined with pulvis antimonialis, and attended him up to the 20th, at which time he was convalescent. He continued to improve up to the afternoon of the 24th, when I was again sent for in great haste to see him. I found him very hoarse; he complained of sore throat, with great difficulty of breathing, and oppression about his chest; I ordered him an emetic of antimonial wine, and cupped him freely over the chest, which relieved him very much for the time. I learned that he thought himself much better that morning and was sitting up and walking about his room; the room being quite warm, he imprudently hoisted a window, and sat opposite it in the draft of air, until he became chilly and hoarse. I visited him the next morning and found the soreness of throat and hoarseness to have increased; I applied a strong solution of the nitrate of silver to the *fauces*, and a blister to his throat, which seemed to afford some relief for a short time; I also ordered that he should inhale the steam from the spout of a teakettle containing warm vinegar and water. It was very evident from the symptoms existing at this visit, that he was laboring under *acute laryngitis*. I was sent for at 2 o'clock, P. M., in great haste, and found my patient very much worse, his face livid and pale, great anxiety of countenance, general restlessness, stridulous breathing, paroxysmal cough, and threatened suffocation. I immediately sent for Dr. Jennings of this city, and on his arrival we agreed on performing the operation of *tracheotomy*. We placed a pillow under the back of the patient's neck, so as to make his throat as prominent as possible, and divided with a scalpel four of the rings of the *trachea*, below the thyroid gland and introduced a canula. The air rushed into the lungs and resuscitated our patient like a *charm;* I say resuscitated, for he was to all appearance in the very act of dying the most agonising death I ever witnessed. The respiration gradually resumed force and frequency, the pulse which before had been intermittent, became steady and increased in frequency. The countenance lost its livid appearance, and the whole surface became covered with a warm perspiration, and consciousness returned. The inflammation of the larynx subsided gradually, the canula remained in the tracha until the 17th of March, at which time I removed it, making twenty-two days from its insertion; I placed a strip of adhesive plaster over the wound, and it healed kindly in a few days.

It is now six years since the operation was performed. I had the satisfaction of seeing Capt. Johnson at my office the other day, and conversed with him respecting his general health, which has been very good ever since he recovered from this attack, although he has never entirely regained the strength and tone of his voice.

Much might be said respecting the pathology of this disease, especially upon the effect produced on the brain and nervous system generally by want of due oxygenation of the *blood;* the difference between the acute and chronic forms of layrngitis, as well as the propriety of the operation; the choice of the operation as it respects that of laryngotomy and tracheotomy, the former by piercing the crico-thyroid membrane with a large sized trochar passing directly into the larynx, and that of tracheotomy, as performed in this case. I would give a decided preference to the operation of *tracheotomy* below the thyroid gland, as it is removed from the seat of the inflammation, which it might augment—but in the chronic form of the disease, or in *croup in children,* I would prefer to pierce the crico-thyroid membrane with a trochar, and introduce a curved canula.

